# A Novel Glucose-Sensitive Scaffold Accelerates Osteogenesis in Diabetic Conditions

**DOI:** 10.1155/2022/4133562

**Published:** 2022-03-18

**Authors:** Ying Jiang, Weihao Li, Congyun Bao

**Affiliations:** ^1^School and Hospital of Stomatology, Kunming Medical University, Kunming 650500, China; ^2^State Key Laboratory of Oral Diseases, West China Hospital of Stomatology, Sichuan University, Chengdu 610041, China

## Abstract

Mandibular bone regeneration is still a big challenge in those diabetic patients with poorly controlled blood glucose. In this study, we prepared a novel glucose-sensitive controlled-release fiber scaffold (PVA-HTCC/PEO-rhBMP2-glucose oxidase (PHPB-G)), which contained the recombinant human bone morphogenetic protein 2 (rhBMP2) by coaxial cospinning and grafted with glucose oxidase (GOD). We presented evidence that PHPB-G could undergo a series of structural changes with the blood glucose and promoted bone regeneration in diabetic rat. PHPB-G expanded the voids in nanofibers when blood glucose levels elevated. More importantly, its slow-release rhBMP2 effectively promoted the healing of bone defects. These data suggested that the PHPB-G delivery system may provide a potential treatment strategy for patients with severe diabetic alveolar bone defects.

## 1. Introduction

Diabetes mellitus is chronic metabolic disease characterized by hyperglycemia. Recent studies reported that diabetes can be associated with oral pathological changes, which are usually ignored in clinic [1]. There is a prediction that 10% of adults would suffer from diabetes in 2030 [2]. In China, the total rate of diabetes or prediabetes is about 24.2% [3]. A couple of diseases including cyst, cancer, and chronic inflammation in maxillofacial often cause the lack of jaw bone. How to promote the bone repairment of diabetic patients is still a big challenge in clinic [4].

Present researches showed that diabetes was closely related to periodontitis [5]. What is more, the risk of periodontitis in diabetic patients is almost three times higher than that in nondiabetic patients [6]. Therefore, the control of blood glucose plays a key role in patients with diabetic periodontitis [7]. Periodontitis destroys periodontal supporting tissue and causes alveolar bone resorption. Hyperglycemia can promote the secretion of inflammatory factors, which result in alveolar bone absorption. That is the main reason for periodontitis patients with tooth loss [8].

In recent years, many natural or synthetic biomaterials, such as chitosan and nanosilica-functionalized scaffolds, proved to be active and beneficial to cure bone defects [9–11]. Electrospun fiber membranes can be loaded with growth factors, such as BMP2, to form sustained-release or controlled-release systems for tissue defect repair [12]. Electrospinning can produce nanofibers, which promote cell adhesion, proliferation, and differentiation by changing the diameter, pore size, and shape of the fibers [13]. However, the traditional electrospinning technology could not respond to the fluctuation of blood glucose rapidly, which result in a low effective drug release. It is urgent to develop glucose-responsive biological scaffolds for the treatment of bone regeneration in patients with diabetic periodontitis.

GOD has been widely used in the delivery system, which has the ability to respond to glucose on a therapeutically relevant time scale [14]. When the concentration of glucose fluctuated, GOD can transform glucose into gluconic acid. As a result, the physical properties of pH-responsive materials altered. Finally, the controlled release of loaded drugs is realized [15]. In the present study, coaxial cospinning electrostatic pinning of nanofiber membrane grafted with GOD (PHPB-G) was utilized for the repairment of bone tissue engineering. The purpose of this study was to provide evidence that PHPB-G delivery system is sensitive to the fluctuation of glucose and can promote the bone reformation by controlling the delivery of rhBMP2, which may hold as novel potential biomaterials for the treatment of diabetic periodontal disease.

## 2. Materials and Methods

### 2.1. Polymeric Membrane Preparation

Glucose and glucose oxidase (GOD) were from Sigma-Aldrich Inc., St. Louis, Missouri, USA. Quaternized chitosan (HTCC, 300 kDa) was from Dongying Tianhua Biomaterial Co., Ltd., China. Recombinant bone morphogenetic protein 2 (rhBMP2) was from Centocor Inc., Pennsylvania, USA. Polyvinyl alcohol (PVA) has a degree of polymerization of 1700 and a degree of alcoholysis of 88%. Polyethylene oxide (PEO, molecular weight: 1 million) was from Chengdu West Asia Chemical Co., Ltd., China.

PVA and HTCC were mixed as shell spinning solution. PEO and rhBMP2 were mixed as core layer spinning solution. To reduce the solubility of the fibers, PVA-HTCC/PEO-rhBMP2 was crosslinked after electrospinning as nanofibers. In short, the fiber membrane was transferred into a vacuum drying oven equipped with glutaraldehyde, in which the glutaraldehyde completely interacted with PVA for 12 hours. Followed by vacuum drying for 3 hours without glutaraldehyde, the fiber membrane was soaked in a solution of toluene diisocyanate (TDI) in dichloromethane (DCM) at a concentration of 0.2 mg/ml for 12 hours. A certain amount of 1-(3-dimethylaminopropyl)-3-ethylcarbodiimide (EDC), N-hydroxysuccinic acid imine (NHS), and GOD (50 *μ*g/30 ml) was dispersed in the aqueous solution. Finally, after being soaked for 12 hours, the fiber membrane was vacuum dried overnight.

### 2.2. Scanning Electron Microscope Analysis

To analyze the cross-sectional shape of PHPB-G, SEM (JeolJSM-400LV, Jeol Ltd., Tokyo, Japan) was utilized to observe the cross-sectional morphology of PHPB with certain concentration glucose solutions (0, 7, 13, and 15 mmol/l) for immersion. Briefly, the samples were put into liquid nitrogen for 20 minutes and then cut to expose the cross section and sprayed with gold. The operating voltage was 20 kV, and the analysis magnification was 500 times and 2000 times, respectively.

### 2.3. Drug Release Study

According to previous studies [16, 17], the evaluation of drug release was applied in the present study. In short, PHPB scaffolds were added into 100 ml of different glucose solutions (0, 5, 17, and 25 mmol/l, dissolved in PBS buffer, pH = 7.4). In order to simulate the fluctuation of blood glucose during the process of drug releasing, glucose or PBS buffer was added into the PHPB scaffold system and observed at certain time, respectively. After being cultured for 0, 0.25, 0.75, 1, 2, 4, 8, 12, 24, 48, 96, and 168 hours, the supernatant of media was collected and centrifuged at 3000 rpm for 10 min. According to the protocol from the manufacture's instruction, the concentrations of supernatant rhBMP2 were then detected by ELISA kit (R&D Systems, Minneapolis, MN, USA).

### 2.4. BMSC Preparation

Bone marrow stromal cells (BMSCs) were obtained from male SD rats (7 days old) in the ways described above [18]. High-glucose Dulbecco's modified Eagle medium (DMEM) contains glucose concentration of 25.5 mmol/l, and low-glucose DMEM contains glucose concentration of 5 mmol/l. In the present study, high-glucose medium was used to mimic the high-glucose culture environment, and low-glucose medium is used to simulate normal blood glucose environment. BMSCs were cultured with 1 ml of medium containing 10% fetal bovine serum (10 nmol/l dexamethasone, 0.1 mmol/l ascorbic acid, and 1 mol/l *β*-glycerophosphate) and 1% penicillin/streptomycin. All the cells were incubated at 37°C under an atmosphere of 5% CO_2_.

### 2.5. Cell Proliferation Assay

According to the protocol, Cell Counting Kit-8 (CCK-8, Dojindo Molecular Technologies, Kumamoto, Japan) was used to evaluate BMSC proliferation. After cocultivation for 1, 3, and 5 days, 100 *μ*l of CCK-8 solution was added into the 96-well plates (1/10^4^ cells/well) at a rate of 10% per well and incubated at 37°C and 5% CO_2_ for 2 h. After three days of cocultivation, detecting with a microplate reader (SpectraMax M5, Molecular Devices, CA, USA), the CCK-8 kit measures cell viability. The optical density value (OD value) of each well was measured at 450 nm.

### 2.6. Immunofluorescence Analysis

Immunofluorescence staining was followed by the methods reported previously [19]. In short, BMSCs were cultured on glass coverslips, fixed in 50% (*v*/*v*) methanol/50% (*v*/*v*) acetone for 5 min, rinsed in DPBS, and incubated with rhodamine tag ghost pen cyclic peptide (F-actin) and FITC labeled OCN (1 : 200 dilution; Abcam, Cambridge, MA, United States) at 4°C overnight. Goat anti-rabbit IgG (Beyotime, Shanghai, China) was incubated for 60 min. DAPI (Solarbio, Beijing, China) counterstained cell nucleus for 10 min. Images were calculated by confocal laser scanning microscope system (Nikon, Tokyo, Japan).

### 2.7. Surgical Procedure

A total of 80 male SD rats (weight 160-180 g) were randomly selected and followed by 4-week high-fat food (60 kcal% fat) feeding; intraperitoneal (i.p.) injection of streptozotocin (35 mg/kg) was performed to induce diabetes SD rat. Fasting blood glucose over 16.6 mmol/l was considered as diabetes ^3^. After the establishment of diabetes, all SD rats were fed a regular rat diet (12.3 kcal% fat) and their blood glucose was monitored regularly. All diabetic rats received bilateral maxillary alveolar bone defect surgery. In short, followed by cutting the skin and muscle tissues along the lower edge of the mandible, peeling off the muscle tissue and periosteum, a circular defect with a diameter of 5 mm in the mandibular corner area was made by a twist drill. The defect area was replaced by PHPB scaffold loaded with or without rhBMP2. To prevent animals from infection, penicillin was utilized for 3 days after scaffold implantation surgery. All the diabetes animals were divided into four groups. The control group was implanted without PHPB scaffolds. The nanofiber group contained no GOD or rhBMP2. The PHPB group had no glucose response but loaded with rhBMP2. The PHPB-G group was implanted with PHPB scaffolds that contained GOD and rhBMP2. After scaffold implantation surgery, the diabetes rats were sacrificed. The maxillary alveolar bones were collected at 4 and 8 weeks, respectively. Alternate slides were stained with Masson staining for histological description. The pictures were captured by an optical microscope (Nikon 80i, Tokyo, Japan) and analyzed by Lane-Sandhu histological scoring standard [16].

### 2.8. Statistical Analysis

All the in vitro tests were performed in triplicate, and the average was used as the results. Data were calculated and shown as mean ± standard deviation. Differences in parameter mean values were analyzed using one-way analysis of variance (ANOVA) test followed by SNK-q multiple comparisons using SPSS software (SPSS 17.0, Chicago, IL, USA). *P* values < 0.05 were considered statistically significant.

## 3. Results

### 3.1. Glucose-Responsive Characterization of PHPB-G

**Figure 1:**
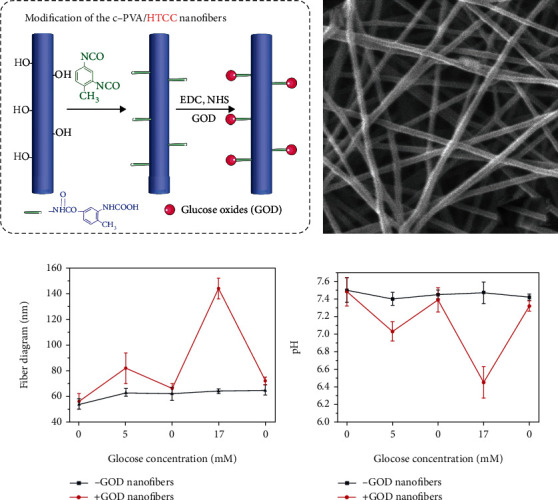
Characterization of PHPB-G. (a) Schematic diagram of crosslinking of PVA-HTCC/PEO-rhBMP2 fiber membrane and immobilization of GOD. (b) Morphology of crosslinked PVA-HTCC/PEO-rhBMP2 fibers. Scale bar = 100 nm (magnification, ×2000). (d) Core-shell structure of PVA-HTCC/PEO-rhBMP2 fiber by TEM. Scale bar = 200 nm (magnification, ×4000). (c) Diameter variation of PHPB fibers grafted with GOD or ungrafted with GOD in different concentrations of glucose solution. (d) pH variation in different concentrations of glucose solution after PHPB fibers were grafted with GOD or ungrafted with GOD soaking.

Since PVA and HTCC dissolved quickly in water at room temperature, the fibers were crosslinked to reduce the solubility in this study. The schematic diagram of PHPB scaffold crosslinked and grafted with GOD is shown in Figure 1(a). The morphology of PHPB after long-term immersion was observed under a scanning electron microscope and transmission electron microscope (Figure 1(b)). The un-crosslinked drug-loaded fibers were staggered into a network with uniform diameter and smooth fiber surface without obvious beading or other defects. The PHPB fibers grafted with GOD showed more sensitivity to glucose concentration than the control group, which means PHPB-G might have the potential ability of glucose-responsive controlled release performance (Figures 1(c) and 1(d)).

### 3.2. Drug Release Performance

**Figure 2:**
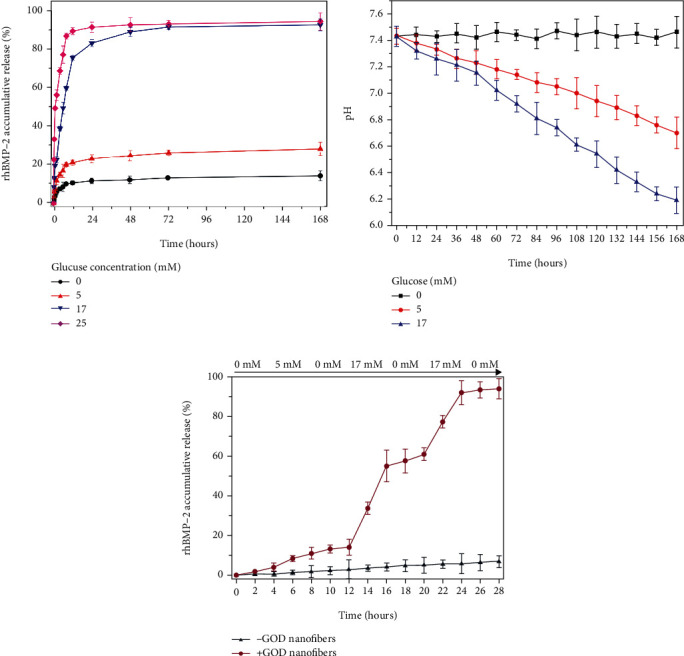
The drug release performance test of PHPB-G. (a) rhBMP2 accumulative release of PHPB-G in different concentrations of glucose solution. (b) pH variation of different concentrations of glucose solution following time. (c) rhBMP2 release dynamics in fluctuate concentration of glucose solution.

When fixed with GOD, the drug release rate of PHPB increased with the growth of glucose (Figure 2). In the first 12 hours, the rhBMP2 released rapidly. The 12-hour accumulative drug release rate was 10.5 ± 1% in 0 mmol/l glucose solution and 21 ± 1.4% in response to 5 mmol/l glucose solution. When immersed in 17 mmol/l glucose solution, the accumulative release rate upregulated to 75.2 ± 1.6%. What is more, it reached 89.4 ± 1.8% in 25 mmol/l glucose solution. Briefly, the drug release rate reached a plateau at 24 hours, and the accumulative release rate of PHPB in 25 mmol/l glucose solution was 91.4 ± 2.7%. There are three main ways of drug release in the glucose response drug release system: drug exudation, fiber degradation, and drug releasing. That might explain why the increased glucose concentration accelerated the rhBMP2 releasing.

### 3.3. Biocompatibility and Osteogenesis of PHPB-G

**Figure 3:**
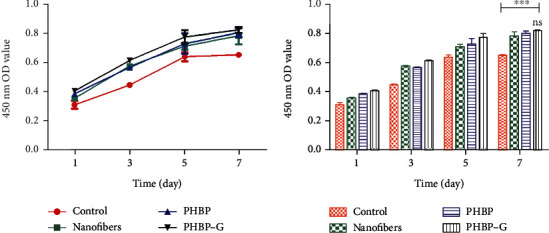
Cell viability of BMSCs cocultured with different materials by CCK-8 in vitro. Data are represented as means ± standard deviation (*n* = 3). ^∗∗∗^*P* < 0.001 when compared with control. ns *P* > 0.05, no significant difference when the PHPB-G group is compared with the PHPB group or the nanofiber group.

**Figure 4:**
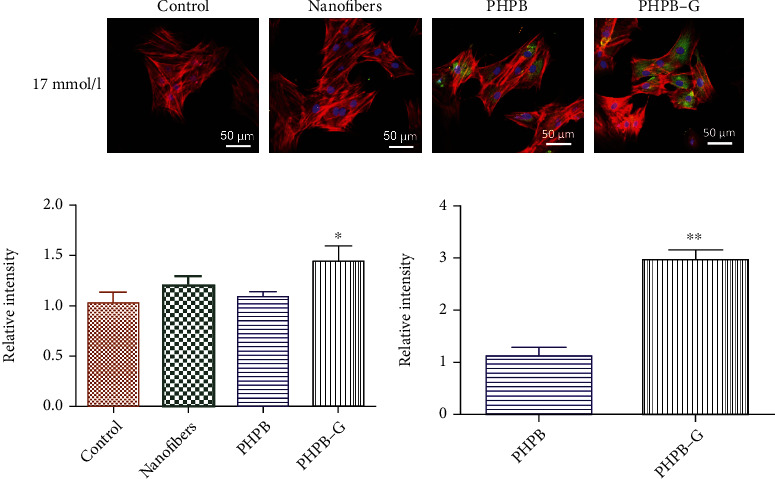
Immunofluorescence analysis to detect the promotion of osteogenesis by PHPB-G. Scale bar = 50 *μ*m (magnification, ×400). (a) The LSCM image of BMSCs cocultured with PHPB and stained with F-actin (red) antibody and FITC-labeled OCN antibody (green). Control group. (b) The relative expression of F-actin. Data are represented as means ± standard deviation (*n* = 3). ^∗^*P* < 0.05 when compared with control. (c) The relative expression of OCN. ^∗∗^*P* < 0.01 when compared with PHPB.

In high-glucose DMEM (17 mmol/l), BMSCs were cocultured with or without PHPB-G scaffold for 7 days. The results of CCK-8 showed that the cell viability of the PHPB-G group was higher than that of the control group (Figure 3). To detect the promotion of osteogenesis by PHPB-G, the cytoskeleton (F-actin) was stained with rhodamine-labeled phalloidin (red), the nucleus was counterstained with TPDI (blue), and OCN (green) was immunofluorescently stained with FITC-labeled OCN antibody after being cocultured 7 days (Figure 4). The results indicated that PHPB-G expressed more F-actin than the control group. What is more, PHPB-G increased the expression of OCN than PHPB when cocultured with BMSCs in high-glucose DMEM.

### 3.4. Alveolar Bone Regeneration of PHPB-G in Diabetic SD Rats

**Figure 5:**
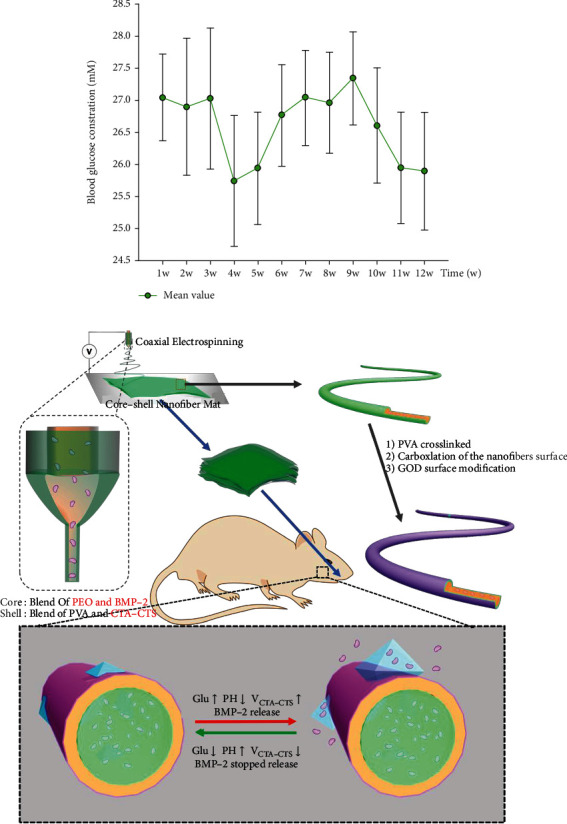
Alveolar bone regeneration of PHPB-G in diabetic SD rats. (a) The average blood glucose concentration of diabetic SD rats. (b) The schematic diagram of fabrication of PHPB-G glucose-sensitive drug-controlled release fiber membrane and its effects on the repairment of mandibular bone defect.

After three days of streptozotocin (STZ) injection, the blood glucose of SD rats was measured with Roche blood glucose meter. When the blood glucose values kept higher than 16.6 mmol/ml, the model was treated as successful. Subsequently, the blood glucose was monitored every week. As shown in Figure 5(a), the blood glucose concentration fluctuated between 25.750 ± 1.022 mmol/l and 27.048 ± 0.675 mmol/l, which indicated that the SD rats maintained diabetic state.

**Figure 6:**
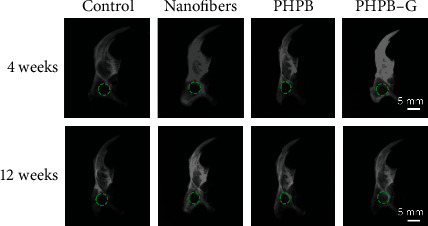
X-ray image of mandibular defect repairment of diabetic SD rats after implanting surgery. Scale bar = 5 mm (magnification, ×40).

**Figure 7:**
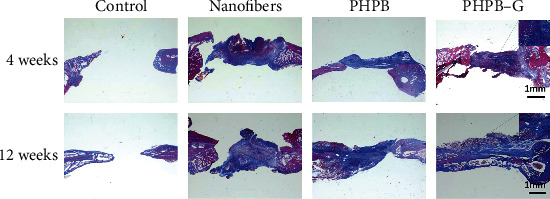
Masson staining for the detection of the inflammatory reaction and new bone regeneration in diabetic SD rats. (d/f) New bone and collagen fiber formation indicated by the arrow. Scale bar = 1 mm (magnification, ×20).

The preparation and function diagram of PHPB-G glucose-responsive drug-loaded releasing system is shown in Figure 5(b). PHPB-G scaffold implantation surgery was carried out to repair the mandibular bone defects. Animals were randomly selected and sacrificed at 4 weeks and 12 weeks after surgery. In the present study, X-ray and Masson staining were utilized to observe the bone tissue repairment by PHPB-G scaffold. The results of X-ray showed that no significant bone reformation was observed at 4 weeks. However, the PHPB-G group showed an increased density in the defect area at 12 weeks (Figure 6). Detected by the Masson staining, inflammatory cells were found around the defect area at 4 weeks (Figure 7). As the inflammatory reaction was involved in the early bone formation, new bone formation can also be found in the PHPB-G group at 12 weeks (Figure 7). Due to the decrease of systemic resistance of diabetes mellitus and the increase of local inflammatory factor secretion in diabetic SD rats, the infiltration of inflammatory cells was observed, which indicated that the inflammatory action time prolonged. However, the PHPB-G group showed an increased capability of new bone reformation particularly in diabetic SD rats.

## 4. Discussion

Environmentally responsive controlled release systems, also known as intelligent controlled-release systems or stimulating controlled-release systems, are synthesized from “smart” materials that can undergo structural or chemical changes in response to external stimuli to achieve drug targeting and regular and quantitative release [20]. In recent years, environmental-responsive controlled release systems have been widely used in biomedical and tissue engineering [21]. Diabetes is a kind of chronic metabolic disease that is widely prevalent all over the world. Long-term high blood glucose causes various histopathological changes including bone tissue [22]. Although some previous studies have reported that the risk of complications could be reduced in well-controlled diabetic patients [23, 24], a large proportion of diabetic patients is still in poor control of blood glucose [25]. In addition, even if some patients have low blood glucose levels, they will be accompanied by some progressive diseases, such as periodontitis and coronary heart disease [26, 27]. In this study, a novel glucose-sensitive drug release system, PHPB-G, was investigated to control the on-demand release of drugs based on glucose fluctuations in diabetic patients.

Compared to the control group, PHPB showed much more active morphological changes when grafted with GOD. It exhibited an expansion trend of nanofiber diameter when immersed in high concentration of glucose solution. What is more, the accumulative release rate of rhBMP2 reached 91.4 ± 2.7% when the glucose concentration increased to 25 mmol/l. By controlling the composition of electrospinning solution and electrospinning technology, nanofiber scaffolds with different diameters, shapes, and drug loading can be formed to control the releasing rate of drugs [28]. Fiber degradation in response to glucose concentration may explain why PHPB-G accelerated the rhBMP2 releasing. All these data suggested that PHPB grafted with GOD could sensitively respond to the changes of glucose levels and control the releasing of rhBMP2 more efficiently.

Chitosan, the most important structure of PHPB-G, has been reported to have positive effects in the reduction of bacterial biofilms, surgical healing of oral wounds, and hardening of teeth [11]. As being loaded with rhBMP2, the result of immunofluorescence staining showed that PHPB increased the expression of F-actin and OCN when cocultured with BMSCs. Particularly, in the PHPB-G group, cell viability and OCN expression showed to be significantly increased than in the control group in high-glucose DMEM culture environment. Ashraf et al. [29] proved that electrospun fiber membrane can be conducive to cell adhesion, proliferation, differentiation, and tissue growth. Park et al. [30] treated recombinant human bone morphogenetic protein 2 (rhBMP2) to have a key role in affecting bone formation. Fennema et al. [31] reported that rhBMP2 was efficient in improving ectopic bone tissue formation in mesenchymal stromal cells (MSCs) and has been widely applied in orthopaedics and dentistry.

In the present study, X-ray showed that no significant bone reformation was observed at 4 weeks, but the density increased in the defect area at 12 weeks. What is more, the bone density of the PHPB-G group increased more than that of the other groups. The secretion of inflammatory factors in diabetic rats was higher than that in normal rats, which indicated that the capability of new bone reformation decreased in diabetic rats. The PHPB-G group showed an increased bone reformation in the present study. The data suggested that PHPB-G might promote the releasing of rhBMP2 in response to high blood glucose. That may explained why the PHPB-G group promoted bone formation more efficiently in diabetic SD rats. The biological scaffolds can carry a variety of biological factors, including TGF-*β*, BMP2, and cellular exosomes [32–34]. However, the true bone remodeling environment in diabetic patients may be much more complex [35]. With the increased bone resorption and reduced bone mass, the risk of fractures increased in diabetes patients [36]. Meanwhile, diabetes affects blood supply and osseointegration of the fracture site [37]. In diabetic patients, the increase of reactive oxygen species and the formation of advanced glycation end products (AGEs) can affect the function of osteoblasts and eventually lead to the decrease of mineral deposition [38]. Mangialardi et al. [39] reported pericyte dysfunction in the bone marrow of type 2 diabetic patients. PHPB-G proved to be sensitive to the fluctuation of blood glucose and controllable to the releasing of active factors, but it still needs a long-term tracking of clinical experiments in further research.

In total, PHPB-G is mainly made up of quaternized chitosan and collagen for the controlled release of rhBMP2. Its honeycombed pore shape and porosity showed advantages in bone regeneration. PHPB-G also exhibits the potential ability of anti-inflammation and promoting osteogenesis on alveolar bone defect. This glucose-sensitive drug delivery system offers a promising method for oral pathological alterations regarding diabetes.

## Data Availability

The data used to support the findings of this study are available from the corresponding author upon request.

## Abbreviations

ANOVA: Analysis of variance
BMSCs: Bone marrow stromal cells
F-actin: Fibrous actin
GOD: Glucose oxidase
HTCC: Quaternized chitosan
OCN: Osteocalcin
PEO: Polyethylene oxide
PVA: Polyvinyl alcohol
rhBMP2: Recombinant bone morphogenetic protein 2
STZ: Streptozotocin.

## References

[1] Cervino G., Terranova A., Briguglio F. (2019). Diabetes: oral health related quality of life and oral alterations. *BioMed Research International*.

[2] Whiting D. R., Guariguata L., Weil C., Shaw J. (2011). IDF diabetes atlas: global estimates of the prevalence of diabetes for 2011 and 2030. *Diabetes Research and Clinical Practice*.

[3] Yang W., Lu J., Weng J. (2010). Prevalence of diabetes among men and women in China. *The New England Journal of Medicine*.

[4] Shao J., Liu S., Zheng X., Chen J., Li L., Zhu Z. (2021). Berberine promotes peri-implant osteogenesis in diabetic rats by ROS-mediated IRS-1 pathway. *BioFactors*.

[5] Mahtani A. A., Jacob C., Lakshmanan R. (2020). Prevalence of diabetes among patients and the assessment of the awareness of the bidirectional relation between diabetes and periodontal disease. *Journal of Family Medicine and Primary Care*.

[6] Papapanou P. N. (1996). Periodontal diseases: epidemiology. *Annals of Periodontology*.

[7] Al-Mubarak S., Ciancio S., Baskaradoss J. K. (2014). Epidemiology and diagnosis of periodontal diseases: recent advances and emerging trends. *International Journal of Dentistry*.

[8] Lalla E., Papapanou P. N. (2011). Diabetes mellitus and periodontitis: a tale of two common interrelated diseases. *Nature Reviews. Endocrinology*.

[9] Armentia S. L., Real J. C. D., Paz E., Dunne N. (2020). Advances in biodegradable 3D printed scaffolds with carbon-based nanomaterials for bone regeneration. *Materials*.

[10] Wang S. J., Jiang D., Zhang Z. Z. (2019). Biomimetic nanosilica-collagen scaffolds for in situ bone regeneration: toward a cell-free, one-step surgery. *Advanced Materials*.

[11] Cicciu M., Fiorillo L., Cervino G. (2019). Chitosan use in dentistry: a systematic review of recent clinical studies. *Marine Drugs*.

[12] Chen Z., Lv Z., Sun Y., Chi Z., Qing G. (2020). Recent advancements in polyethyleneimine-based materials and their biomedical, biotechnology, and biomaterial applications. *Journal of Materials Chemistry B*.

[13] Hong J., Yeo M., Yang G. H., Kim G. H. (2019). Cell-electrospinning and its application for tissue engineering. *International Journal of Molecular Sciences*.

[14] Volpatti L. R., Matranga M. A., Cortinas A. B. (2020). Glucose-responsive nanoparticles for rapid and extended self-regulated insulin delivery. *ACS Nano*.

[15] Lee I., Cheon H. J., Adhikari M. D. (2020). Glucose oxidase-copper hybrid nanoflowers embedded with magnetic nanoparticles as an effective antibacterial agent. *International Journal of Biological Macromolecules*.

[16] Li H., Liao H., Bao C., Xiao Y., Wang Q. (2017). Preparation and evaluations of mangiferin-loaded PLGA scaffolds for alveolar bone repair treatment under the diabetic condition. *AAPS PharmSciTech*.

[17] Chen Y., Tang C., Zhang J., Gong M., Su B., Qiu F. (2015). Self-assembling surfactant-like peptide A6K as potential delivery system for hydrophobic drugs. *International Journal of Nanomedicine*.

[18] Kim K., Dean D., Wallace J., Breithaupt R., Mikos A. G., Fisher J. P. (2011). The influence of stereolithographic scaffold architecture and composition on osteogenic signal expression with rat bone marrow stromal cells. *Biomaterials*.

[19] Li W., Zhao S., Yang H. (2019). Potential novel prediction of TMJ-OA: MiR-140-5p regulates inflammation through Smad/TGF-*β* signaling. *Frontiers in Pharmacology*.

[20] Huang B., Chen F., Shen Y. (2018). Advances in targeted pesticides with environmentally responsive controlled release by nanotechnology. *Nanomaterials*.

[21] Zhu C. L., Wang X. W., Lin Z. Z., Xie Z. H., Wang X. R. (2014). Cell microenvironment stimuli-responsive controlled-release delivery systems based on mesoporous silica nanoparticles. *Journal of Food and Drug Analysis*.

[22] Popp J., Waters D. L., Leekity K. (2017). Using the Centers for Disease Control and Prevention’s stay independent checklist to engage a community of American Indians and raise awareness about risk of falls, 2016. *Preventing Chronic Disease*.

[23] UK Prospective Diabetes Study (UKPDS) Group (1998). Effect of intensive blood-glucose control with metformin on complications in overweight patients with type 2 diabetes (UKPDS 34). *Lancet*.

[24] Skyler J. S., Bergenstal R., Bonow R. O. (2009). Intensive glycemic control and the prevention of cardiovascular events: implications of the ACCORD, ADVANCE, and VA Diabetes Trials: a position statement of the American Diabetes Association and a Scientific Statement of the American College of Cardiology Foundation and the American Heart Association. *Journal of the American College of Cardiology*.

[25] Huang E. S., Liu J. Y., Moffet H. H., John P. M., Karter A. J. (2011). Glycemic control, complications, and death in older diabetic patients: the diabetes and aging study. *Diabetes Care*.

[26] Tonna S., El-Osta A., Cooper M. E., Tikellis C. (2010). Metabolic memory and diabetic nephropathy: potential role for epigenetic mechanisms. *Nature Reviews. Nephrology*.

[27] Yong T. Y., Phillipov G., Phillips P. J. (2007). Management outcomes of patients with type 2 diabetes: targeting the 10-year absolute risk of coronary heart disease. *The Medical Journal of Australia*.

[28] Shahhosseininia M., Bazgir S., Joupari M. D. (2018). Fabrication and investigation of silica nanofibers via electrospinning. *Materials Science & Engineering. C, Materials for Biological Applications*.

[29] Ashraf R., Sofi H. S., Malik A., Beigh M. A., Hamid R., Sheikh F. A. (2019). Recent trends in the fabrication of starch nanofibers: electrospinning and non-electrospinning routes and their applications in biotechnology. *Applied Biochemistry and Biotechnology*.

[30] Park S. Y., Kim K. H., Kim S., Lee Y. M., Seol Y. J. (2019). BMP-2 gene delivery-based bone regeneration in dentistry. *Pharmaceutics*.

[31] Fennema E. M., Tchang L. A. H., Yuan H. (2018). Ectopic bone formation by aggregated mesenchymal stem cells from bone marrow and adipose tissue: a comparative study. *Journal of Tissue Engineering and Regenerative Medicine*.

[32] Nassif L., El Sabban M. (2011). Mesenchymal stem cells in combination with scaffolds for bone tissue engineering. *Materials*.

[33] Poongodi R., Chen Y. L., Yang T. H. (2021). Bio-scaffolds as cell or exosome carriers for nerve injury repair. *International Journal of Molecular Sciences*.

[34] Ghandforoushan P., Hanaee J., Aghazadeh Z. (2022). Novel nanocomposite scaffold based on gelatin/PLGA-PEG-PLGA hydrogels embedded with TGF-*β*1 for chondrogenic differentiation of human dental pulp stem cells _in vitro_. *International Journal of Biological Macromolecules*.

[35] Conti F., Wolosinska D. T., Pugliese G. (2013). Diabetes and bone fragility: a dangerous liaison. *Aging Clinical and Experimental Research*.

[36] Li G., Prior J. C., Leslie W. D. (2019). Frailty and risk of fractures in patients with type 2 diabetes. *Diabetes Care*.

[37] Choi Y. J., Chung Y. S. (2016). Type 2 diabetes mellitus and bone fragility: special focus on bone imaging. *Osteoporos Sarcopenia*.

[38] Suzuki R., Fujiwara Y., Saito M. (2020). Intracellular accumulation of advanced glycation end products induces osteoblast apoptosis via endoplasmic reticulum stress. *Journal of Bone and Mineral Research*.

[39] Mangialardi G., Ferland-McCollough D., Maselli D. (2019). Bone marrow pericyte dysfunction in individuals with type 2 diabetes. *Diabetologia*.

